# The impact of dengue viruses: Surveillance, response, and public health implications in Queensland, Australia

**DOI:** 10.1016/j.puhip.2024.100529

**Published:** 2024-07-04

**Authors:** Alan Silburn, Joel Arndell

**Affiliations:** Western Sydney University, Campbelltown, 2560, NSW, Australia

**Keywords:** Dengue virus, Communicable diseases, Surveillance systems, Public health policy, Queensland, Global impact

## Abstract

This study examines dengue transmission, symptoms, vaccination efforts, treatment options, and global impact, focusing on Australia, especially Queensland. It evaluates current surveillance and response systems, identifies areas for improvement, and proposes strategies to enhance public health preparedness. Highlighting the socioeconomic impact of dengue outbreaks, the study underscores the need for integrated public health measures, effective vaccines, advanced surveillance methods, and sustainable mosquito control programs to mitigate the threat of dengue outbreaks and potential endemicity.

## 1 Introduction

Since it was first reported in China as early as the 3rd Century during the Chin Dynasty, dengue viruses impact approximately two and a half billion people worldwide and are now considered a major global public health challenge [[1], [2], [3]]. Specifically in the tropic and sub-tropic nations, Dengue has surged in prevalence largely due to the exponential population growth rate, changes in climate, unplanned urbanisation, ineffective mosquito control programs, and a lack of public healthcare facilities [1]. Like others, the northern parts of Australia have experienced outbreaks of Dengue virus requiring acute attention.

The study aims to comprehensively examine Dengue viruses, encompassing their transmission, symptoms, vaccination, treatment, and global impact. With a specific focus on Australia, particularly Queensland, its objectives include assessing the effectiveness of current Dengue surveillance and response systems, identifying areas for improvement, and proposing strategies to enhance public health preparedness and response. Additionally, the study aims to raise awareness about the increasing risk of Dengue due to factors such as climate change and urbanisation. Its scope extends to analysing data on outbreak occurrences, seasonality trends, and geographic distribution while considering broader contextual factors influencing Dengue transmission. Ultimately, the study seeks to provide insights for strengthening surveillance, prevention, and control measures to mitigate the growing threat of Dengue outbreaks and endemicity.

## 2 Dengue virus overview

Dengue viruses are a member of the genus *Flavivirus* of the family *Flaviviridae* and are an arthropod-borne virus spread by the *Aedes* mosquito [1]. The Dengue virus includes four serotypes (DENV1-4) depending on the differences in surface protein structure [1]. The transmission dynamics of dengue depend on a variety of factors, including the density of mosquitoes, the prevalence of previous dengue infections, and the climate. Dengue virus gains entry into the host organism through the skin following the bite of a DENV-infected mosquito. Once transmission occurs, the Dengue virus binds to its particular receptors on the surface of a host cell before initiating receptor-mediated endocytosis [2]. Once access into the cell is permissible, the viral particle is uncoated, and the RNA is released in a host cell where it directs the synthesis of viral proteins. In only a few hours after the initial infection occurs, tens of thousands of copies of the viral molecules are produced leading to systemic compromise characterized by increased vascular permeability, plasma leakage, haemorrhagic manifestations, and thrombocytopenia [2].

The World Health Organization classifies dengue fever into two groups based on the symptoms experienced by the patient. Classic or uncomplicated dengue fever can present with either mild fever only or encompass more incapacitating characteristics including a sudden onset of high fever, severe headache, retro-orbital pain, myalgia, arthralgia or rash [4]. Dengue haemorrhagic fever (DHF) or severe dengue fever however can become life-threatening due to a transient increase in vascular permeability resulting in plasma leakage, haemorrhage, thrombocytopenia and haemoconcentration [4]. Of the four serotypes (DENV1-4), DENV-2 is associated with more severe dengue disease as evidenced by a significant association with DHF when compared to DENV-1. In addition, DENV-2 and DENV-3 are reported as being twice as likely to result in DHF than DENV-4 [5,6]. This is concerning regarding prevalence as DENV2 serotypes remain dominant followed by DENV-4 serotypes [7].

The socioeconomic impact of dengue outbreaks on affected communities is profound and multifaceted. Beyond the immediate health burden, dengue imposes substantial economic costs on individuals, families, and healthcare systems. Direct costs include medical expenses for treatment, while indirect costs stem from productivity losses due to illness and caregiving responsibilities. Furthermore, dengue outbreaks can exacerbate existing social inequalities, disproportionately affecting vulnerable populations with limited access to healthcare and sanitation services. In addition to individual hardships, dengue outbreaks can disrupt local economies, particularly in tourism-dependent regions, as public health concerns and travel advisories deter visitors [8]. Addressing the socioeconomic impact of dengue requires a holistic approach, integrating public health measures with strategies to strengthen healthcare systems, improve sanitation infrastructure, and mitigate the social determinants of health [9].

## 3 Vaccination and treatment

Although DENV has been highlighted as a global public health concern, to date, there is no vaccine for the primary prevention of initial dengue infection for Australians. Since 1928, different methods of vaccine production and delivery methods have been trialled, the first being made from filtrates of suspensions both of dengue-infected mosquitoes and of the dried blood of dengue patients however failed to demonstrate prophylactic value [10]. Since, multiple DENV vaccines have undergone different phases of clinical trials, three of which recently have shown promising results; Dengvaxia®, TV003, and TAK-003 [11]. At present, Dengvaxia® is the only licenced vaccine available on the global market following clinical trials, however, the availability and indications for use vary. Challenges noted by scientists in the development of a single vaccine include establishing one that provides coverage for all four serotypes whilst limiting vaccination failure rates in the periods following administration [11].

In Australia, although available, Dengvaxia® is not indicated for the primary prevention of the virus. The Australian Technical Advisory Group on Immunisation (ATAGI) states that vaccination with Dengvaxia® should only be considered for Australians on very rare occasions when all of the following conditions are met: the patient is aged 9–45 years; has had previous dengue infections; are intending to reside in highly dengue-endemic regions for an extended period; and only if the potential benefits are deemed to outweigh the risks [12].

Supportive symptomatic treatment can assist a patient infected with dengue fever such as the administration of antipyretics to reduce fevers and analgesics to reduce headaches and joint pains. Likewise, dehydration can be prevented by encouraging oral rehydration therapy, however, if oral intake is not tolerated, an intravenous fluid replacement can be used to prevent hypovolemic shock in infected patients [2]. For patients with dengue haemorrhagic fever, platelet transfusions are usually given to patients who have low platelet counts, although the exact platelet count at which platelets should be administered is poorly defined [13].

## 4 Global impact of dengue

Over the last twenty years, there has been a significant rise in the prevalence of dengue worldwide, presenting a significant public health concern. Between 2000 and 2019, the World Health Organization (WHO) observed a tenfold increase in reported cases, soaring from 500 000 to 5.2 million. 2019 witnessed an unparalleled peak, with reported incidents spreading across 129 nations, particularly concerning the tropic and sub-tropic nations [14]. Since 1960, reports of dengue virus in the tropic and sub-tropic nations of Southeast Asia, the Pacific and the Americas have surged in prevalence accounting for more than 83.6 % of the overall reported outbreaks accompanied by the equivalent mortality and social burden [15]. This can largely be attributed to the exponential population growth rate, changes in climate, unplanned urbanisation, ineffective mosquito control programs, and a lack of public healthcare facilities [1,2]. Following a modest decrease in cases between 2020 and 2022, attributed to the impact of the COVID-19 pandemic and reduced reporting, there has been a resurgence of dengue cases globally in 2023. This resurgence is marked by a notable escalation in the quantity and scope of outbreaks, as well as their simultaneous occurrence in regions previously untouched by dengue [14]. Worryingly, it has been approximated that three and a half billion people, or 40 % of the world's population, reside in these dengue-endemic regions with a mortality rate surpassing 5–20 % [1].

## 5 Dengue in Australia

Dengue is not endemic in Australia however widespread outbreaks have been documented previously across eastern Australia, from northern New South Wales to northern Queensland [16]. Since 1990, regular outbreaks have occurred, the largest observed occurring in Cairns of far northern Queensland during 2008–2009 with more than a thousand reported cases. This outbreak overwhelmed the capacity of the local public health response unit to contain further transmission [16]. Despite increased public health control efforts in North Queensland, dengue outbreaks have become more frequent over the last two decades suggesting a heightened risk of endemicity [17].

From January 1999 to December 2010, Australia documented a total of 5853 dengue cases, encompassing both locally acquired and imported cases. Queensland accounted for the majority of cases (53.0 %), followed by New South Wales (16.5 %). The highest frequency of dengue outbreaks (54.2 %) was observed between 2008 and 2010. Notably, during this period, the data suggests an association with dengue incidence with overseas arrivals (29.9 %), households equipped with rainwater tanks (33.9 %), Indigenous population (27.2 %), separate houses (26.5 %), terrace house types (26.9 %), and economically disadvantaged individuals (42.8 %) [18].

Between 2012 and 2022, 13 343 dengue cases were reported; an increase of 231 % compared to the previous decade [19]. The largest outbreak was noted in 2016, when there were over 2000 confirmed cases, including many from Western Australia, Victoria and New South Wales. More recently, between January 2023 and January 2024, there were a total of 1117 dengue cases reported, marking a 1.7-fold increase compared to the five-year rolling mean of 645.6 cases for the same period [20].

## 6 Surveillance in Australia

In Australia, the dengue virus is listed as a mandatory reportable disease. All DENV cases are required under the Public Health Act 2005 to be notified to the primary health department within each state or territory to be processed under the National Notifiable Disease Surveillance System (NNDSS) following laboratory confirmation of DENV infection [21]. Managed by the Australian Government under the auspices of the Communicable Diseases Network Australia (CDNA), the NNDSS is a passive surveillance system framework that collates, analyses and disseminates information on communicable diseases to the greater Australian public [22]. Similar to other Australian states, Queensland Health submits data on dengue notifications detailing the possible exposure and infection dates, the number of active and inactive cases, the source of infections by either autochthonous or overseas acquired, and the geographical place of residence of the notified cases at a statistical local area level [21]. By maintaining a complete array of communicable disease data at the local, state, and national levels, the National Notifiable Disease Surveillance System is able to identify if spatial or temporal patterns emerge as to action and limit further disease progression and transmission. While the NNDSS framework is functional, it is important to acknowledge the limitations and challenges associated with dengue surveillance. Like with other epidemiological models, underreporting of cases and data quality issues can impact the accuracy of the information collected, potentially leading to gaps in understanding DENV transmission dynamics.

Of the data that Queensland Health submits, key sources can be identified as crucial to optimising a public health defence against the dengue virus. Firstly, reporting the calendar date of a possible exposure or confirmed infection is of key significance as DENV, like all other vector-borne diseases, is highly influenced by seasonality [23]. Seasonality in vector-borne diseases is a well-known epidemiological phenomenon largely because *Aedes* mosquitoes are highly influenced by meteorological and environmental conditions [16]. In Australia, dengue transmission is noticeably confined to a certain geographical profile that allows optimum vector breeding, however, DENV cases in these areas are witnessed to reduce or become absent during the dry season months through winter and spring [16]. This prompts concern that the currently changing climate may increase the geographic range and scale of DENV outbreaks in Queensland and the greater nation made apparent by the interstate outbreak in 2016.

Climate change has likewise influenced DENV transmission by impacting the vector growth and development, and availability of habitat and can alter the virus characterises such as reducing the length of the extrinsic incubation period. Significant correlations have been witnessed between weather and climate variability and the prevalence of dengue including the distinct seasonal variants that occur during El Niño Southern Oscillation. The potential impact of climate change on DENV epidemiology has been extensively analysed predicting an expansion in the geographic distribution of dengue virus if climate change continues [16].

New technologies and surveillance methods can help mitigate the impact of DENV outbreaks. Predictive modelling and digital surveillance platforms, for instance, provide opportunities to improve early detection, response, and monitoring of dengue outbreaks. Similar technologies and methods were demonstrated successfully during the COVID-19 pandemic whereby self-reporting and contact tracing systems became the social norm [24]. By integrating these technologies into current surveillance systems, we can better identify emerging trends and target control efforts more effectively. This ultimately leads to stronger public health responses against dengue in Australia.

## 7 Intervention areas of focus

Implementing interventions for combating the spread of the dengue virus in Queensland presents both feasibility and practical challenges. Raising public awareness about the increasing risk of dengue virus is essential, especially with changing meteorological and environmental conditions. This involves educating the population about preventive measures before peak risk times during wet seasons. While public health campaigns can effectively disseminate information, ensuring widespread awareness and subsequent action from the population requires significant resources and sustained effort. Additionally, accurately targeting high-risk populations within Queensland adds complexity to the awareness-raising efforts, as different regions may have varying levels of susceptibility to dengue outbreaks due to factors like population density and environmental conditions. seasons.

Secondly, utilising communicable disease surveillance for targeted interventions is a practical approach to mitigate the risk of dengue transmission. However, conducting geographical investigations and implementing targeted preventative actions require robust surveillance systems and coordination among various stakeholders. Challenges may arise in accurately identifying the primary sources of disease transmission, particularly in areas with complex transmission dynamics or limited resources for surveillance activities. Nevertheless, successful disease surveillance programs in other regions, such as the Centres for Disease Control and Prevention (CDC) in the United States and the European Centre for Disease Prevention and Control (ECDC), demonstrate the effectiveness of strategic surveillance in controlling communicable diseases and informing public health interventions [25].

Thirdly, implementing mosquito surveillance and control measures is a practical strategy to reduce the prevalence of the dengue virus but requires sustained effort and resources. Successful examples from other regions include vector control programs in countries like Singapore and Brazil, which have effectively reduced mosquito-borne disease transmission through comprehensive surveillance, community engagement, and innovative control strategies like Wolbachia-infected mosquito releases [26]. At present, public health units in northern Queensland are actively engaged in educating and supporting the population to break the transmission cycle through mosquito control measures. Under the Queensland Dengue Management Plan 2015–2020 [27], areas identified for mosquito control activities are mapped from respective addresses determined from contact tracing. These areas are subsequently targeted for the deployment of lethal ovitraps in large arrays to combat adult mosquito populations and by removing or mosquito-proofing water-bearing containers to mitigate against larval distribution [27]. This methodology is common across a broad array of public health approaches in the fight against vector-borne diseases, however, challenges such as resource constraints, public compliance, and the emergence of insecticide-resistant mosquito strains can hinder the effectiveness of these measures [28,29]. Likewise, under the legislation set forth by the Australian Government and outlined within the Public Health Regulation 2005, local governments require Queensland residents and land occupiers to control mosquito breeding on their properties and maintain compliance with their water storage containers. Additionally, the Director-General of Queensland Health or the Chief Executive Officer of a local government can authorise an Approved Inspection Program or an Authorised Prevention and Control Program under which authorised persons may enter premises for the purposes of monitoring regulatory compliance to public health directives [27].

Addressing the hesitancy of government parties to implement legislative travel restrictions or bans for high-risk geographical destinations presents ethical and practical challenges. In Queensland, the dengue virus is attained by either autochthonous or overseas-acquired transmissions [21]. For autochthonous transmission, DENV is initiated when local *Aedes* mosquitoes bite an infectious individual before passing the virus on to a new host within the same geographical setting [30]. Overseas-acquired DENV occurs when an individual becomes infected with the dengue virus outside of Australian borders before returning in an infectious state. Often for vector-borne disease, a combination of both transmission processes can occur as an infectious returned traveller can instigate the autochthonous dispersion of the specific disease [30]. While travel restrictions can effectively contain epidemics, they raise concerns about individual rights, economic impacts, and international relations. Implementing targeted travel advisories and quarantine measures may be more feasible alternatives to blanket travel bans, balancing public health priorities with economic and social considerations. Examples from past epidemics, such as the Ebola outbreak in West Africa, demonstrate the effectiveness of targeted travel advisories and quarantine measures in limiting disease spread while minimizing disruptions to global travel and trade [31]. For the Australian population, advice about the communicable diseases and health risks of global destinations is published on the publicly accessible ‘Smart Traveller’ website that is curated by the Australian Government Department of Foreign Affairs and Trade [32]. Overall, addressing the feasibility and practical implications of interventions to combat the dengue virus requires a multi-faceted approach, informed by successful examples from other regions and tailored to the specific context and challenges faced in Queensland.

In addition, involving the public health sector in addressing industries contributing to climate change represents a proactive approach but may encounter resistance from sectors prioritising economic interests over public health concerns. Engaging industries in sustainable practices to mitigate climate change requires strong governmental regulations and incentives, which can face opposition from powerful stakeholders. However, successful examples from other regions demonstrate the feasibility of such interventions. For instance, initiatives in European countries like Sweden and Germany have effectively involved multiple sectors in reducing greenhouse gas emissions through policies promoting renewable energy and sustainable practices in transportation and industry. These examples highlight the importance of governmental leadership and collaboration across sectors in addressing climate change, which in turn can mitigate the risk of vector-borne diseases like dengue [33].

## 8 Conclusions

Dengue virus remains a significant global public health challenge, with increasing prevalence and impact worldwide. This study comprehensively examined Dengue viruses, focusing on transmission, symptoms, vaccination, treatment, and global impact, with a specific emphasis on Australia, particularly Queensland. Key findings indicate a rise in Dengue cases in Australia, with Queensland experiencing regular outbreaks, suggesting a heightened risk of endemicity [16,17]. The study highlights the socioeconomic impact of Dengue outbreaks, emphasizing the need for holistic approaches integrating public health measures with strategies to strengthen healthcare systems and mitigate social determinants of health.

Recommendations for future research and policy development include enhancing Dengue surveillance and response systems, identifying areas for improvement, and proposing strategies to enhance public health preparedness and response. Future studies should focus on developing effective vaccines covering all four DENV serotypes, improving surveillance methods using predictive modelling and digital platforms, and implementing sustainable mosquito surveillance and control measures. Additionally, further research is needed to understand the impact of climate change on Dengue transmission dynamics and to address the feasibility and practical implications of interventions to combat Dengue virus.

The implications of study findings for public health practice and policy in Queensland, Australia, and beyond are significant. Policymakers should prioritise the implementation and enforcement of existing public health policies, such as mandatory reporting of dengue cases, isolation, and travel bans while adapting them to respond to changing epidemiological patterns. Adequate resource allocation is essential to strengthen surveillance, research, and control efforts, particularly in the face of increasing dengue outbreaks and the potential for endemicity. Regional and global collaboration is crucial for sharing knowledge, best practices, and resources to address the global burden of dengue effectively. Additionally, efforts should focus on health equity, ensuring that marginalised and vulnerable populations receive targeted interventions to reduce disparities in dengue burden and improve overall public health outcomes. By implementing these recommendations and considering the implications of the study findings, policymakers and public health practitioners can work towards reducing the burden of dengue and safeguarding communities' health and well-being.

## Author statement

This study did not require ethical approval as it involved a retrospective analysis of publicly available and anonymized data, with no direct involvement of human subjects.

## Declaration of competing interest

The authors declare that they have no known competing financial interests or personal relationships that could have appeared to influence the work reported in this paper.
